# Adaptation of Bird Communities to Farmland Abandonment in a Mountain Landscape

**DOI:** 10.1371/journal.pone.0073619

**Published:** 2013-09-02

**Authors:** João Lopes Guilherme, Henrique Miguel Pereira

**Affiliations:** Centro de Biologia Ambiental, Faculdade de Ciências da Universidade de Lisboa, Lisboa, Portugal; Università degli Studi di Napoli Federico II, Italy

## Abstract

Widespread farmland abandonment has led to significant landscape transformations of many European mountain areas. These semi-natural multi-habitat landscapes are important reservoirs of biodiversity and their abandonment has important conservation implications. In multi-habitat landscapes the adaptation of communities depends on the differential affinity of the species to the available habitats. We use nested species-area relationships (SAR) to model species richness patterns of bird communities across scales in a mountain landscape, in NW Portugal. We compare the performance of the classic-SAR and the countryside-SAR (i.e. multi-habitat) models at the landscape scale, and compare species similarity decay (SSD) at the regional scale. We find a considerable overlap of bird communities in the different land-uses (farmland, shrubland and oak forest) at the landscape scale. Analysis of the classic and countryside SAR show that specialist species are strongly related to their favourite habitat. Farmland and shrubland have higher regional SSD compared to oak forests. However, this is due to the opportunistic use of farmlands by generalist birds. Forest specialists display significant regional turnover in oak forest. Overall, the countryside-SAR model had a better fit to the data showing that habitat composition determines species richness across scales. Finally, we use the countryside-SAR model to forecast bird diversity under four scenarios of land-use change. Farmland abandonment scenarios show little impact on bird diversity as the model predicts that the complete loss of farmland is less dramatic, in terms of species diversity loss, than the disappearance of native Galicio-Portuguese oak forest. The affinities of species to non-preferred habitats suggest that bird communities can adapt to land-use changes derived from farmland abandonment. Based on model predictions we argue that rewilding may be a suitable management option for many European mountain areas.

## Introduction

Changes and loss of biodiversity can directly influence ecosystem structure and functioning [1], reduce ecosystem resilience to disturbances such as global warming [2], and jeopardize vital ecosystem services that support human well-being [3]. Currently, land conversion is recognized as the main factor driving global biodiversity change [4].

In Europe, during the last decades, agricultural intensification and industrialization of former extensively managed arable lands have promoted land abandonment and marginalization of many remote mountain areas [5]. This socio-ecological trend is mostly driven by human migration to urban areas [6], reflects the generalized demand for better life conditions (namely material well-being; [7]) and exhibits high chances of irreversibility [8]. The reduction of use or the complete abandonment of farmland has had a profound impact on the dynamics of many mountain landscapes. As land is abandoned, vegetation disturbance is highly reduced and secondary succession takes place, allowing the regeneration of native vegetation. The secondary expansion of shrubs and the regeneration of forest on former farmland and pastures lead to a simplification of the traditional landscape mosaic [5], [9], which affects regional biodiversity [10], [11].

Responses to farmland abandonment vary across and within taxonomic groups [12]–[15]. Interestingly, the development of forest in former farmland may not necessarily favour all forest specialist taxa. Evidence exists that some forest beetle and bat species [e.g., 16, 17], benefit from certain traditional management practices that restrain excessive vegetation closure and maintain open areas. In birds, the diversity of performed ecological functions [18] and the differential affinity of species to different habitats underlie the wide range of responses to farmland abandonment observed within this group: while some species suffer detrimental effects, other species increase their abundance [9], [15], [19]–[21]. The observed trends also differ between regions. For instance, as agricultural intensification in lowlands increases, uplands may be the only remaining suitable grounds for open-habitat bird species [21]. At the same time, rewilding of traditional farmland in mountain areas may bring advantages such as improved ecosystem services [22], [23], and increasing bird species richness as a function of forest development towards climax [19], [20], [24]. Birds are vital mobile links for maintaining ecosystem function [25], acting as ecosystem service providers at the genetic, resource and process levels [18]. Consequently, it is crucial to understand how landscape dynamics affect bird diversity patterns in order to understand, and remediate, the dramatic declines of bird populations registered across Europe during the last decades (see PECBM – Pan-European Common Bird Monitoring scheme: http://www.ebcc.info/pecbm.html, and references therein).

Species-area relationships (SAR) constitute a valuable framework to study biodiversity patterns. Nested SAR are curves constructed by estimating mean richness across sampled subplots within larger areas, that assist in understanding the processes underlying patterns of biodiversity across scales [26]. However, SAR have been used preferably at large spatial scales, to estimate the biodiversity of large regions [27], since at small scales, habitat heterogeneity is a major determinant of diversity patterns [28]. For example, bird diversity is highly influenced by habitat diversity. In order to accommodate this habitat effect, several studies proposed the incorporation of the multi-habitat context in the SAR framework [29]–[32]. The countryside-SAR model proposed by Pereira and Daily [31] is unique in considering that different species groups differentially use extant set of habitats in a given area.

In spite of being a powerful tool to study species diversity patterns [33], nested SAR focus only in numerical species gains and they do not explicitly consider the loss of species in additional sampled area [34]. The composition of species assemblages between two areas changes through various processes derived from species traits (e.g., dissimilar dispersion strategies) and landscape/regional characteristics (e.g., diversity of habitats and their spatial configuration) [35]–[36]. Therefore, the difference in species composition between two areas, or the species similarity decay (SSD) with distance, is a fundamental aspect of species spatial patterns [35]–[36] and should be taken into account in landscape and regional scale studies. The SSD constitutes a good surrogate to understand beta-diversity patterns of species groups complementing the SAR analyses.

Following the tendency observed within many European mountain areas [5], the Peneda-Gerês Mountains (NW Portugal) have been subject to farmland abandonment during the last decades, which led to a land-use alteration across the region’s landscape. In this study we aim at predicting the effects of current (and possible future) landscape transformations on species richness patterns of bird communities in the Peneda-Gerês Mountains. We predict that bird species use multiple habitats in the landscape and can adapt to land-use change caused by farmland abandonment. To address this prediction we analyse classic and multi-habitat SAR at the landscape level and SSD at the regional level. Specifically, we ask the following questions: (i) what are the bird diversity patterns in the different land-uses at the landscape scale? (ii) does species composition similarity decays with distance at the same rate for different land-uses at the regional scale? (iii) are species richness patterns better described by classic or by (multi-habitat) countryside-SAR model? (iv) what are the consequences of different land-use change scenarios for the regional bird communities?

## Methods

### Ethics Statement

Permission to access privately owned land was given by all the land owners. This study did not require any approval for animal care and use because it was an observational field study, not involving the capture and handling of wild animals nor their maintenance in captivity.

### Study Region

**Figure 1:**
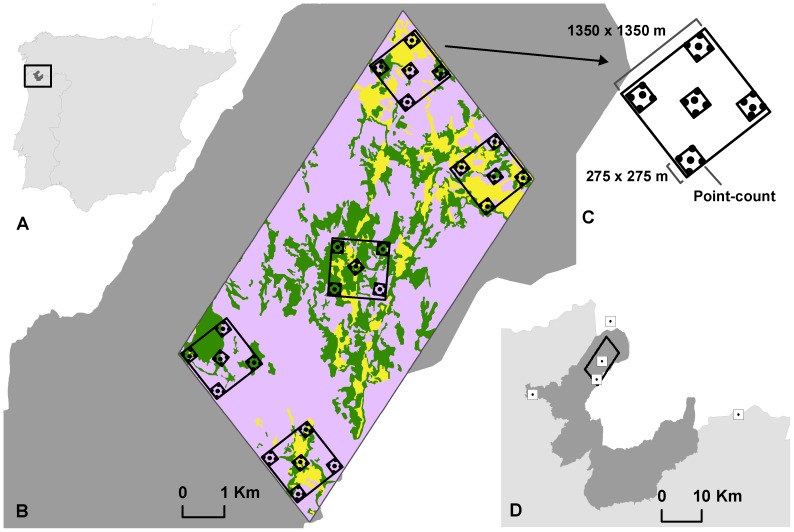
Map of the study region. A: Location of the study region in Northern Portugal (dark-grey area represents the Peneda-Gerês National Park); B: Nested sampling scheme and the three land-uses considered in the study: farmland (yellow), shrubland (rose) and oak forest (green); C: Detail of a local-square; D: Distribution of the local-squares used for the species similarity decay analysis.

Our study region consists of the Peneda-Gerês National Park (PNPG), NW Portugal (Figure 1A). The region encompasses the Peneda and Gerês Mountains covering an area of 69 592 ha of extensively managed woodland-pasture-agriculture mosaic. The region is located in the transition between the Mediterranean and Eurosiberian biogeographic zones in the proximity of the Atlantic coast. Topographic relief is complex with a high plateau, slopes with various bedrocks and narrow valleys, with an elevation ranging from 300 m to 1340 m.

The core area of our study is the landscape of the Castro Laboreiro Valley (ca. 42°01’N, 8°09’W) (Figure 1B) which covers 4725 ha. Formerly harbouring a self-sufficient community based on agriculture and pastoralism, this area has been characterized by a marked rural exodus since the 1960’s that triggered the abandonment of traditional agricultural practices. Albeit this trend, agriculture is still the main economic activity in the region. Most of the land is privately owned but some areas are communal and mainly used for pasture.

### Land-Use Characterization

The definition and categorization of the different land-uses was based on available land-use maps for Portugal (IGEOE: http://www.igeoe.pt) and Galicia (SITGA: http://sitga.xunta.es). Similarly to other European mountain landscapes, our study region has a complex structure composed by a large set of natural and semi-natural habitats that resulted from the anthropogenic modification of the natural landscape. All these habitats were grouped into three main land-use categories: farmland, shrubland and oak forest. In this paper we use habitat and land-use interchangeably.

*Farmland*: nowadays few fields are used for crops and most farmland is occupied by semi-natural pastures used for cattle grazing or fodder production. Some vegetable patches and fruit trees are maintained. Human made structures (villages and scattered houses) were included in this category.

*Shrubland*: this broad category includes areas dominated by heaths (*Erica sp.*), gorses (*Ulex sp.*) and *Genista tridentata*, and areas of tall shrublands of brooms (*Cytisus sp*.), gorses and heaths. Some of these areas also include bedrocks and/or dispersed trees.

*Oak Forest*: the native Galicio-Portuguese oak forests of *Quercus robur* and *Q. pyrenaica* constitute the climax vegetation of the region. Although the area of Galicio-Portuguese oak forest is much reduced relative to its biogeographic potential, in the PNPG there are extremely well preserved patches. Within the Castro Laboreiro Valley, native oak forests represent 92% of the forested area, with the remnant area corresponding to small pine plantations of *Pinus sylvestris* and *P. pinaster*, and scattered patches of other natural broadleaved species. Therefore, the total area of forest was included in this category, although bird data were only collected in oak forests.

The region is clearly dominated by shrubland which, acting as the matrix land-use in the landscape, represent 73% of the study area (Figure 1B). Farmland and oak forest are equally represented, accounting respectively for 12% and 15% of the Castro Laboreiro Valley’s landscape.

### Bird Data and Experimental Design

Bird data were obtained from 30 m fixed-radius point-counts (approximately 0.3 ha) [37]. We set our sampling unit to 0.3 ha due to the particular fine-grained aspect of the Castro Laboreiro Valley landscape, as 30% of the agricultural fields in our study area are smaller or equal to 0.3 ha [38]. Although 0.3 ha may be small relatively to the territories of some open area bird species, we believe that all species occurring in farmlands were effectively sampled. Point-counts were visited once by the same observer (JLG) to avoid between-observer variations, during the breeding season of 2009 (from late April to mid-June). All the birds heard or seen in a ten-minute period were recorded. No counts were performed under strong wind, rain or cold weather. Birds of prey, nightjars and owls, and aerial feeders (swifts and swallows) were excluded from the statistical analysis, as this survey method is not adequate for these groups [39]. Juvenile birds were also excluded from the analysis.

In order to study SAR at the landscape scale point-counts were set according a nested sampling scheme (Figures 1B, 1C): point-counts (approximately 0.3 ha) were aggregated in groups of five forming the centre and corners of a 275×275 m plot (approximately 7.56 ha), such that within each plot the minimum distance between point-counts was 152 m (distance from centre to corner point-counts); five such plots form the centre and corners of a 1375×1375 m local-square (approximately 189 ha); finally, five local-squares form the centre and corners of a landscape polygon (4725 ha) corresponding to the Castro Laboreiro Valley. We assume that breeding and foraging territories of the species used in our analysis are within the Castro Laboreiro Valley landscape unit.

We studied SSD based on five 1375×1375 m local-squares placed in the study region according to a gradient of distance (0, 5, 10, 20 and 40 km). The central and the 5 km distant local-squares were the same as used in the SAR study, whilst three additional local-squares were placed 10, 20 and 40 km from the centre of the landscape (Figures 1C, 1D).

Local-squares were placed strategically to have variable representation (percentage) of each land-use, being the number of point-counts in each local-square stratified according to the area of each land-use category. Our experimental design totals 200 point-counts distributed in the region; however, two point-counts were excluded since they were inaccessible. We surveyed 54 point-counts in farmland, 76 in shrubland and 68 in oak forest.

### Species Groups Description

For the definition of species groups by their habitat affinity we performed a correspondence analysis (CA) using data from all the 198 surveyed point-counts (i.e. regional scale). The Levins index (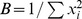, where *x_i_* is the relative abundance of each species (individuals/point-count) in land-use category *i*, in relation to species total abundance across the three land-use categories [40]), was used as a measure of habitat breadth to sort generalist species from specialists.

**Figure 2:**
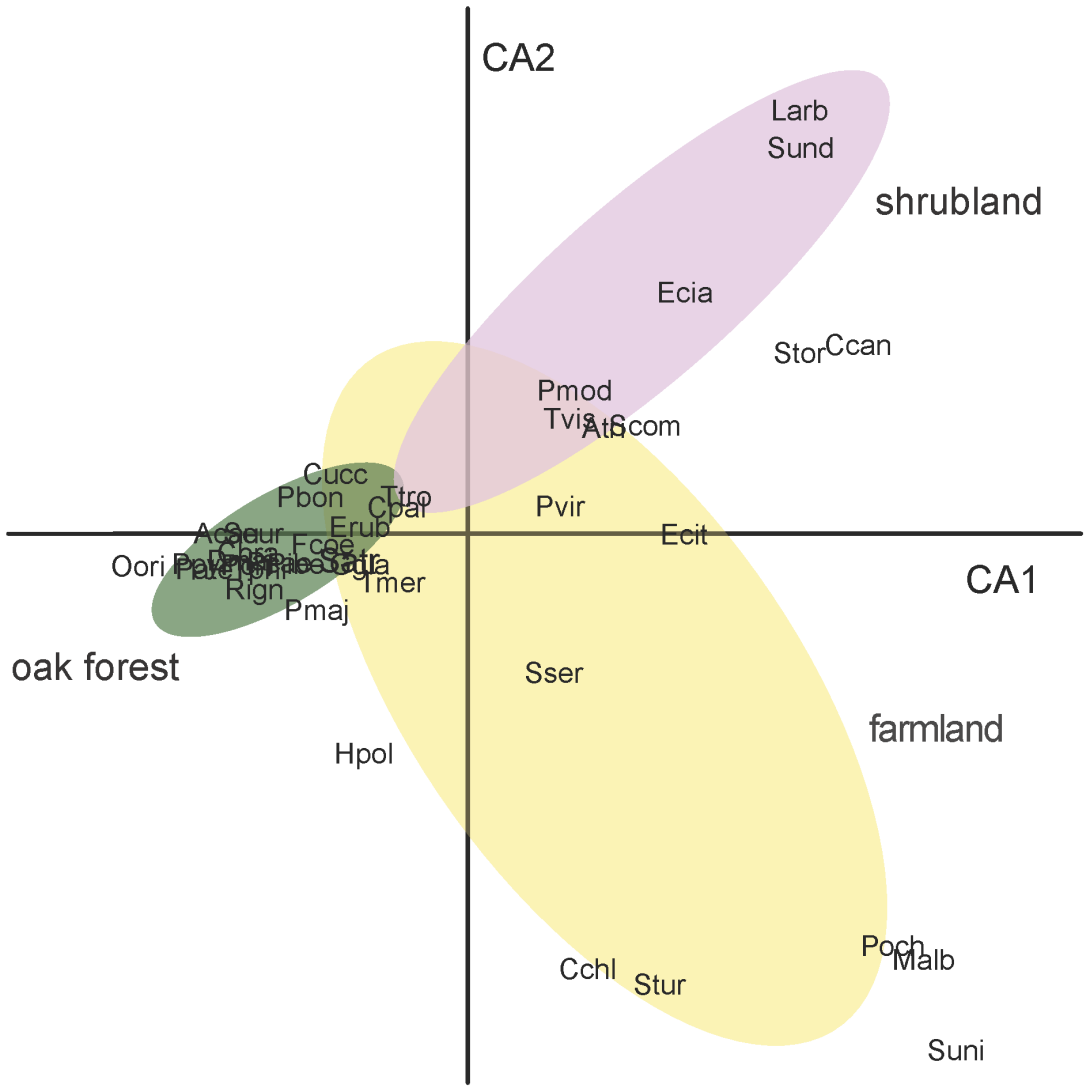
Plot of first two axes of the correspondence analysis of bird relative abundance versus point-count. The ellipses enclose the points of the same land-use (confidence region of 95%): farmland (yellow), shrubland (rose) and oak forest (green). Bird species are represented by their species code (see Table S1).

The correspondence analysis was robust (15.3% of explained variation) in identifying the species associated with the three land-uses (Figure 2). The first axis (CA1, exp. variance = 8.1%; eigenvalue = 0.64) distinguishes oak forest from shrubland, while the second axis (CA2, exp. variance = 7.2%; eigenvalue = 0.57), discriminates farmland. Based on the CA outputs and the habitat breadth calculated for each species (Table S1), four bird species groups were identified: three groups were considered habitat specialists (farmland, shrubland and forest species) while the species equally distributed across land-uses (i.e., with a wide habitat breadth) formed a fourth group of generalists. Of the 43 bird species found, 10 were classified as farmland species, 7 as shrubland species, 16 as (oak) forest species and the remnant 10 were considered generalists (Table S1).

### Diversity Patterns Analysis

For studying bird diversity patterns at the landscape scale we analysed species-area patterns. Classic and countryside SAR curves were fit to data using non-linear regressions. For the classic-SAR we used the power model: 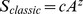, where the number of species *S* (response variable), grows with sampled area *A* (predictor variable), influenced by *c* and *z*, two parameters that are dependent on the taxonomic group and the sampling scheme respectively [41]. The classic-SAR of total species and of each species group were fitted by adding average species richness values from presence-absence data recorded in all sampling units within the landscape (i.e. point-counts), accumulating data from 0.3 ha to 7.56 ha, 189 ha and 4725 ha (curve type IIIA, *sensu* [21]). We assumed that the nested clusters of 0.3 ha point-counts are appropriate for sampling each scale (e.g., 7.56 ha plots were sampled by five 0.3 ha point-counts in the centre and corners of the plot, and in turn each 189 ha local-square was sampled by grouping five plots corresponding to 25 point-counts, see Figure 1B). Classic-SAR of total species and each species group were also obtained for each land-use. In either case, curves were fitted using only points-counts sampled in each of the land-uses in relation to the habitat area cover in every 7.56 ha plot, 189 ha local-square and 4725 ha landscape.

**Figure 3:**
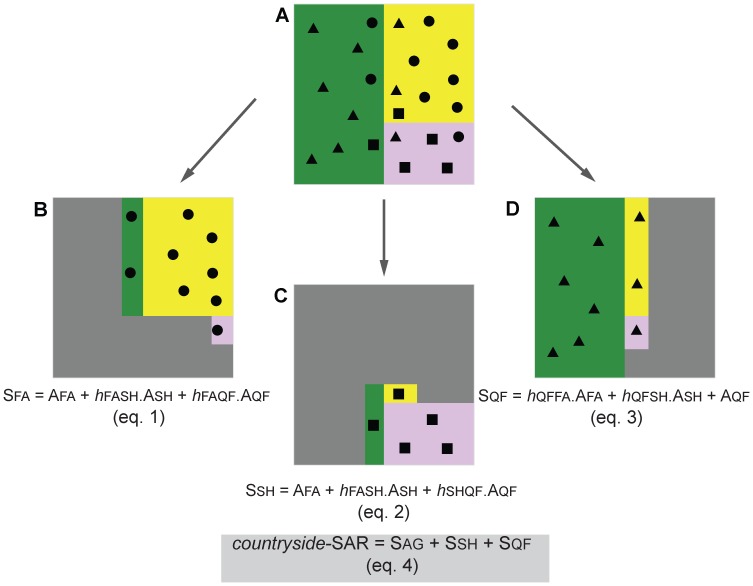
Schematic representation of the countryside-SAR model. The countryside-SAR estimates the total number of species in the landscape accounting for a differential use of habitats by different species groups. The conceptual landscape (A) is composed by different proportions of farmland (FA; yellow), shrubland (SH; rose) and oak forest (QF; green); this landscape is used by farmland (dots), shrubland (squares) and forest (triangles) species. For each species group (B, C and D) the model estimates the affinity of the group *h*, to each of the habitats; the affinity can be interpreted as the proportion of area of each habitat that can be effectively used by a species group (eq. 1, 2 and 3 respectively). The total number of species in the multi-habitat landscape is given by the sum of the number of species in each group (eq. 4).

In order to consider the multi-habitat context of the landscape the countryside-SAR model was fitted to data set. The countryside-SAR model accounts for the differential use of habitats by different species groups, with species groups characterized by species with similar habitat preferences (i.e. affinity) [31]. Thus, the number of species in each group *S_i_* (response variables), depends on the raw affinity 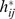 of the group *i* to habitat *j*, with *A_j_* (predictor variables) representing the area of that habitat: 
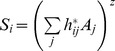
. A non-linear regression was performed for each species group, estimating the raw habitat affinities 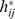. Next we normalized the habitat affinities by dividing each estimated affinity by the maximum estimated affinity: 
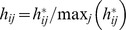
. The normalized affinities can be interpreted as the proportion of area of each habitat that can be effectively used by a species group (Figure 3). For comparison to the classic-SAR, we re-wrote the model as 
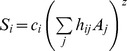
, with 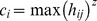. The total number of species *S*, present in the multi-habitat landscape is given by the sum of the number of species in each group where *m* is the number of species groups: 
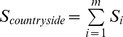
. Countryside-SAR curves were fitted for all species and for each species group in each land-use and in the entire landscape. The fit of both classic and countryside models to the data set was evaluated using the corrected Akaike information criterion (
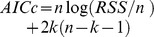
, where *n* is the number of data points and *k* is the number of parameters in the model, including the estimated variance).

Species similarity decay (SSD) was studied at the regional scale by comparing the slope of the relationship (simple linear regression) between the turnover of species and the distance between samples [42]. Species turnover was measured using Sørensen index: 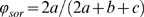, where *a* refers to the number of shared species in samples A and B, and *b* and *c* refers to the species solely found in samples A and B respectively. The index was calculated for all pairwise comparisons at the point-count scale for total diversity, for intra-habitat diversity and for each species group in the intra-habitat context. All the analyses were performed in R 2.15.2 environment [43].

### Scenarios of Land-Use Change

The estimated countryside-SAR model for the total number of bird species in the Castro Laboreiro Valley landscape was used to project the number of bird species in the landscape under four scenarios of land-use change for the PNPG. Although the scenarios were based in previous studies [7], [44], they represent idealized situations. We assume the area lost by a habitat is replaced in equal proportions by the other two habitats [33]. The story lines for the four scenarios and details on land-use transitions are given in Table 1. Scenario 1 assumes the steadily abandonment of agriculture as human population ages, with the progressive homogenization of the landscape, due to the replacement of farmland by shrubland associated with early succession stages [38], and to the increase of native oak forest [7]. Scenario 2, assumes a dramatic depopulation of the study area leading to the nearly complete abandonment of agriculture in the study area (we considered a reduction of farmland to 1% of the study area) accompanied by progressive rewilding of the landscape as native oak forest matures and expands. Under this scenario, landscape management targets the re-establishment of ecological processes at the landscape scale, envisioning nature conservation and ecosystem services enhancement [44]. Scenario 3 assumes a reversal of the current population patterns as a consequence of the return of people to farming activities. A renewed society, concerned with the environment, would adapt innovative techniques to traditional farming knowledge foreseeing high quality farming products [7]. The area of farmland would increase such as the area managed by each farmer. Finally, scenario 4 assumes that a global crisis could lead to a dramatic increase of farmlands and agricultural intensification for high production of direct goods, with the dramatic decrease of oak forest (we considered a reduction to 1%), as a function of clearing for agriculture or its substitution by exotic forest plantations.

**Table 1:** Story lines and proportion of area covered by each land-use of four land-use scenarios for the Peneda-Gerês National Park.

	Land-use proportion (%)		
Story line	Farmland	Shrubland	Oak Forest
**Present**			
	12	73	15
**Scenario 1**			
Persistence of agricultural abandonment due to population ageing and emigration.Development of shrublands in farmland. Progressive homogenization of landscape fromsecondary succession.	6	76	18
**Scenario 2**			
Dramatic depopulation leads to a virtual end of agricultural activity. Dramatic loss (nearly total)of farmland. Rewilding of landscape as native oak forest regenerates and matures.	1	78.5	20.5
**Scenario 3**			
Return of new generations to countryside and farmland activities. Innovative techniquesare applied to traditional farming envisioning high-quality products, biodiversity conservationand ecosystem services enhancement. Increase of farmland area and size of farmholds.	16	76.5	7.5
**Scenario 4**			
Agricultural intensification and mechanization for the production of average quality direct goods.Increase of farmland area and size of farmholds. Clearing of most native forest and hedgerowsand possible forestation with fast-growing exotic trees.	19.5	79.5	1

## Results

**Figure 4:**
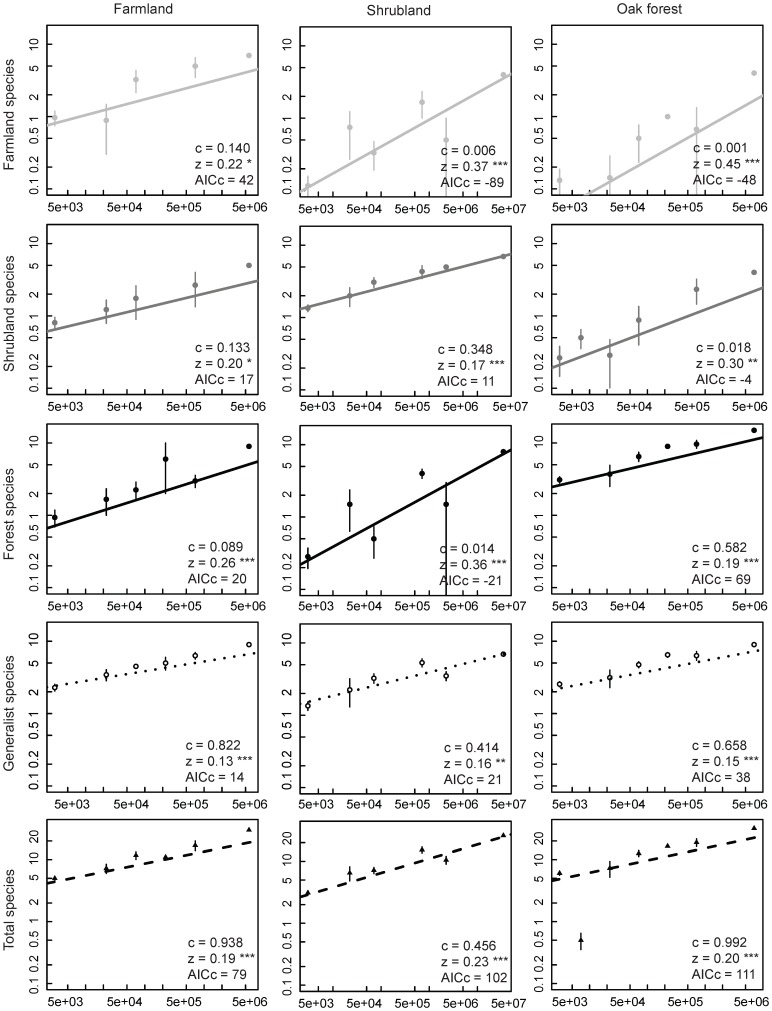
Plots of classic-SAR of each species group and total species in the three land-uses. Species-area relationships are represented in the Log-Log space for each species group (farmland, shrubland, forest and generalist species) and total species in each land-use (farmland, shrubland and oak forest). Symbols represent mean species number for each area category; error bars represent ±1 standard error. Parameters *c* and *z* are given for all the SAR; p-values of all regressions are shown with *z* parameter (p<0.05*, <0.01**, <0.001***). The fit of the model to the data is given by corrected Akaike Information Criteria (AICc).

We found significant species-area relationships for all species groups in each of the three land-uses (Figure 4). Species richness of each specialist group, as given by the *c*-values of tested SAR, was highest for the favourite land-use of the group. Generalist species had the highest *c*-value in farmland, but also showed relatively high values in oak forest and shrubland. The *z*-values of classic-SAR suggest a lower degree of spatial turnover of each specialist species group in its favourite habitat within the landscape compared to the other land-uses (Figure 4).

At the regional scale we found stronger species similarity decay in farmland and shrubland than in oak forest (Table 2). In farmland this pattern is primarily due to the decay of generalist and shrubland specialists, as farmland specialists do not display a pattern of regional species turnover (p-value n.s.). Moreover, farmland species also show non-significant relationships between turnover and distance in shrubland and oak forest. Shrubland, in spite of supporting lower number of species, show high SSD at the regional scale, due to the variation of shrubland specialists and generalist species. Oak forest, although exhibiting a much smoother pattern of total SSD compared to farmland and shrubland, display a significant turnover with distance of generalists and forest specialists within the region (Table 2).

**Table 2:** Regional species similarity decay of each species group and of the total species in the three land-uses.

	Farmland		Shrubland		Oak forest	
Species group	Intercept	slope	Intercept	slope	Intercept	slope
Farmland	1.97E-01	n.s.	2.22E-02	n.s.	4.17E-02	n.s.
Shrubland	2.44E-01	−4.6E-06***	2.41E-01	−3.45E-06***	1.22E-02	n.s.
Forest	1.62E-01	−1.9E-06**	4.15E-02	n.s.	2.75E-01	−1.43E-06***
Generalist	4.12E-01	−4.5E-06***	1.43E-01	−2.22E-06**	4.96E-01	−2.11E-06***
Total species	3.35E-01	−3.4E-06***	2.07E-01	−3.07E-06***	3.88E-01	−1.71E-06***

p-values of all regressions are shown with slope: * = p<0.05; ** = p<0.01; *** = p<0.001; n.s. = non-significant).

When considering the multi-habitat context of the landscape, the results for the two tested models were different (Table 3). The *c*-values were much higher when the affinities of each species group to the different habitats were considered. Differences were very marked, with *c*-values of farmland and shrubland specialists two times higher when estimated by the countryside-SAR, and even higher for forest specialists. On the other hand, *z*-values were similar when estimated by both models. The affinities of each specialist species group, as estimated by the countryside-SAR, have maximum values in the respective preferred habitat, while generalist species showed similar preference for farmland and forest grounds. The countryside-SAR model was the best model (based upon AICc) to describe the data for each species group (Table 3). The countryside-SAR also had a much better fit for the total bird species richness compared to the classic-SAR (respectively 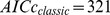 and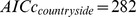).

**Table 3:** Classic and countryside species-area relationships in the multi-habitat context of each species affinity group in the multi-habitat landscape.

	Estimated parameters					Model fit
Speciesgroup	c	z	hAG	hSH	hQF	AICc
Farmland						
classic-SAR	0.028	0.329	–	–	–	46
countryside-SAR	0.089	0.299	1	2.70E-04	2.10E-03	−8
Shrubland						
classic-SAR	0.184	0.219	–	–	–	68
countryside-SAR	0.359	0.182	3.60E-02	1	1.00E-03	50
Forest						
classic-SAR	0.197	0.257	–	–	–	228
countryside-SAR	0.574	0.216	3.30E-03	7.80E-06	1	134
Generalists						
classic-SAR	0.518	0.178	–	–	–	142
countryside-SAR	0.723	0.168	9.10E-01	1.20E-02	1	114

Parameters h_AG_, h_SH_ and h_QF_ represent the affinity of the species group for farmland, shrubland and oak forest respectively. The countryside-SAR model has better fit to the data set in all analyzed relationships as shown by the corrected Akaike information criteria (AICc).

**Figure 5:**
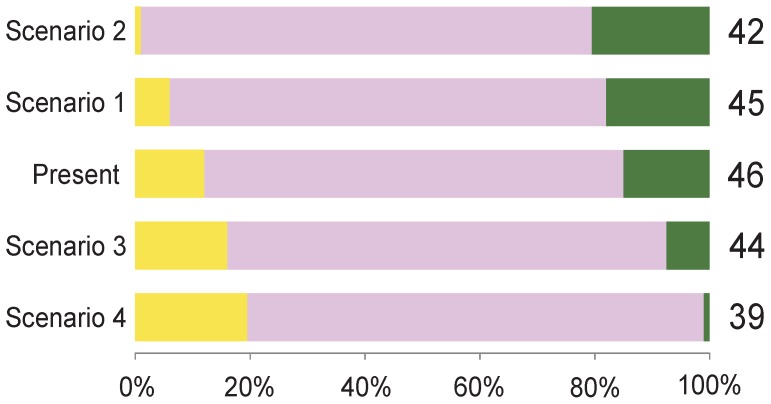
Current and projected number of bird species using the countryside-SAR under four land-use scenarios. Bars show the representation of each land-use in the Castro Laboreiro Valley for four scenarios of land-use change. For each scenario the number of bird species is given as projected by the countryside-SAR model: farmland (yellow), shrubland (rose) and oak forest (green).

We used the countryside-SAR model to project the number of bird species that can be found in the Castro Laboreiro Valley according to different land-use scenarios (Figure 5; Table 1). Since the classic-SAR does not account for the multi-habitat context, the total number of species in the landscape would be the same (48 species) under the four scenarios. On the other hand, the countryside-SAR model forecasts different numbers of bird species as a consequence of landscape transformation, by considering the different conservation value of the available habitats and the affinity of the species groups to each habitat (Figure 5).

## Discussion

### Bird Diversity Patterns

Bird richness and diversity were similar in farmland and native oak forest but lower in shrubland. There was a considerable overlap of species groups across the three land-uses, with about one-third of the species generalists, few specialist species (i.e., with narrow habitat breadth), and even fewer exclusive species (Figure 2; Table S1). In Peneda Mountains agricultural fields are isolated and native oak forest is still fragmented into small patches embedded in a shrubland matrix [38]. We suggest that the observed species overlap across land-uses is a consequence of the highly fragmented nature of the landscape because fine-grained landscapes improve the connectivity of habitats which allows more specialists to be found outside their preferred habitat. Compared to several other taxa, bird diversity is primarily influenced by landscape-scale heterogeneity, due to their dispersal ability [10]. Birds actively choose which habitats to explore and connect the habitats by actively moving in the landscape [25]. Moreover, in farmland-forest systems bird distributions may be influenced by interactions between distance to edge, habitat selection and dependence on shrubs [45]. For example, forest specialists such as coal tit *Parus ater*, crested tit *P. cristatus*, firecrest *Regulus ignicapilla* and short-toed treecreeper *Certhia brachydactyla*, were observed on farmland, actively using the live sedges of broadleaved trees and shrubs that act like corridors connecting forest patches [46]. As to farmland species, like tree pipit *Anthus trivialis*, landscape fragmentation increases their occurrence in forest patches due to edge effects [47]. In the case of plants, Proença and Pereira [33] found that the degree of species overlap between habitats in Peneda Mountains was much lower compared to birds. They found larger differences in the affinity values (given by the countryside-SAR) between the preferred and the alternative habitats of each species group than what we report here for birds, probably because the habitats are characterized by dominant plant formations [48].

Fragmented heterogeneous landscapes may favour generalist species and limit the landscape scale diversity of specialists: first, there can be a saturation of the species pool at smaller scales in fragmented landscapes than in larger forest ecosystems [33], as forest bird diversity is a function of forest matureness, growing differentiation [19], [24] and continuous area [46], [47]; and second, as shrubland develop into early stage forest, the community of birds may range only from generalist farmland species to forest specialists [15].

Still, at the landscape scale all species groups responded positively to increasing areas of their preferred habitat with classic-SAR *z*-values between 0.17 and 0.22. Surprisingly even higher *z*-values were found for each species group in their non-preferred habitats (Figure 4). However, these higher *z*-values do not necessarily correspond to a higher species turnover of specialists in non-preferred habitats, as the regional SSD of specialists is not consistently higher in their non-preferred habitats relatively to their preferred habitats (Table 2). We hypothesize, instead, that this pattern is driven mainly by a sampling effect: at small scales specialist species go undetected (note the low *c*-values of specialists in their non-preferred habitat; Figure 4), but at larger scales the infrequent use of non-preferred habitats by these species can be detected [41].

### Species-Area Relationships and the Multi-Habitat Context

Species-area patterns of total species and of each species group in the Castro Laboreiro Valley landscape were better described by the countryside-SAR model. The better performance of the countryside-SAR emphasizes the role of habitat heterogeneity as a key descriptor of species richness, as landscape composition and area are correlated and both contribute to species richness [41]. The dramatic loss of one habitat does not imply the complete disappearance of the species associated to that habitat, as species may survive in the landscape using alternative habitats depending on their affinity to each habitat [31]. Different authors have proposed modifications to the classic-SAR models in order to include various habitats. The models proposed by Tjørve [29] and Triantis et al. [30] account for the multi-habitat landscape, differing in that the former suggests the combination of multiple species-area curves (i.e. of different habitats) to describe species diversity, and the latter, i.e. the *choros* model, assumes the number of existing habitats in a given area, but ignores the available surface of each habitat. Although both models consider the role of habitat heterogeneity, they do not consider that different taxa use the available habitats differently. Recently, Koh and Ghazoul [32] proposed the matrix-calibrated model, which partitions the *z*-value of the power model in two components: a constant that describes the complete unsuitability of the matrix to the analysed taxa, and a parameter that represents the sensitivity of the taxa to modified habitats. The extinction risk of endemic birds across 20 biodiversity hotspots was better predicted by the matrix-calibrated model compared to the classic-SAR and the countryside-SAR [32]. However, the authors did not discriminate species groups and assumed the same affinity to human-modified habitats for all species analysed, which is an inadequate assumption to test the countryside-SAR model, because different species adapt and persist differently in the landscape after habitats are subject to land-use alteration [31]. The countryside-SAR estimates the affinity of selected taxa to the available habitats, and hence gives more accurate understanding of the impacts of land-use change on avian and other taxa diversity. Many species have the potential to use and adapt to different habitats. However, many biological attributes (e.g., minimum population size, migratory strategy, habitat breadth) that give key information to understand the variation of species responses to land-use change [15], are not accounted for in the countryside-SAR model. Nonetheless, the model has the potential to adequately forecast the bird dynamics derived from the ongoing rural abandonment dynamics common to most European mountain areas.

### Land-use Change and Conservation Implications

Land-use scenario analyses stress the role played by native oak forest and farmland in sustaining bird diversity in the Castro Laboreiro Valley. High bird diversity is sustained along a gradient of loss and gain of these land-uses, and higher richness could be expected through the expansion of both habitats. According to the countryside model, the nearly complete loss of farmland or native oak forest results in the loss of bird diversity (scenarios 2 and 4, Figure 5). However, it does not imply the complete disappearance of the species associated to the receding habitat, as species may survive in the landscape using alternative habitats depending on their affinity to each habitat. The outputs of these extreme situations, however, are different, suggesting that the reduction of farmland is potentially less dramatic in terms of species richness loss compared to the clearing of native Galicio-Portuguese oak forest. In addition, this projection may be conservative because the return to agriculture would imply the clearing of hedgerows for mechanization facilitation, while these natural corridors are important to maintain woodland connectivity and woodland species diversity [46]. Our predictions are in accordance with our results and agree with the theoretical predictions by Navarro and Pereira [23], suggesting that natural habitats may host as much species diversity as farmland, and birds can adapt to land-use change caused by farmland abandonment. Two processes may explain these results: first, open-area species may use and persist in alternative natural habitats that mimic farmland (e.g., in forest clearings), compared to forest species that require more vegetation structured habitats. Nonetheless, narrow farmland specialists could become locally extinct (e.g., skylark *Alauda arvensis*, due to improved negative forest edge effects [45]), contributing to a simplified bird community. Second, the community of farmland specialist birds may already be simplified. This hypothesis is supported by scenario 3 (Figure 5; Table 1) which projects a gain of species resulting from farmland expansion.

Agricultural intensification is the main driver of the widespread bird declines observed in Europe [49]. Nonetheless, many studies advocate negative impacts from land abandonment on bird communities and call for the development of agri-environment schemes to preserve upland extensive farming systems [15], [19]–[20]. Perhaps this approach is the main reference for European conservationists (and land stakeholders [44]) because true wilderness areas no longer exist in Western Europe. However, there is little evidence that agri-environment schemes are broadly successful and feasible in the long-term [50], [51]. Several authors are therefore suggesting alternative land management strategies, such as rewilding [23], [52].

The rewilding of mountain landscapes undergoing farmland abandonment through secondary forest regeneration can bring benefits with regard to particular ecosystem services (e.g., carbon storage, high quality timber and water cycle regulation [6], [22]) and biodiversity conservation [22], [23]. Forest bird specialists would benefit more from rewilding and forest spread than farmland birds. Nonetheless, having intermediate characteristics between the Atlantic and the Mediterranean climates, Galician-Portuguese native oak forests have the potential to harbour highly diverse bird communities. The occurrence of natural and human disturbances (such as wildfires and grazing [19], [21]) should result in sufficient heterogeneity of successional stages of forest dynamics within the landscape. As such, rewilding may maintain enough patches of open habitat, which are important to the persistence and for the dispersal of open-habitat bird species [53], especially so for specialists of Mediterranean origin [20].

## Supporting Information

Table S1 **List of the bird species recorded during point-counts in the study region.** For each species is indicated the species code, land-uses where the species was recorded, habitat breadth and species affinity group: FA – farmland species, SH – shrubland species, QF – forest species, Gn – generalist species.
(DOCX)
