# Supplementary material for: Adaptation of Bird Communities to Farmland Abandonment in a Mountain Landscape
Source: PLoS One. 2013 Sep 2;8(9):e73619. doi: 10.1371/journal.pone.0073619 (PMC3759457; doi:10.1371/journal.pone.0073619)
Supplement: Table S1 — List of the bird species recorded during point-counts in the study region. For each species is indicated the species code, land-uses where the species was recorded, habitat breadth and species affinity group: FA – farmland species, SH – shrubland species, QF – forest species, Gn – generalist species. (DOCX) [file pone.0073619.s001.docx]

**Supporting Information**

**TABLE S1** **List of the bird species recorded during point-counts in the study region.**

| **Species name** | | **Code** | **Used habitats** | **Habitat breadth** | **Species group** |
| --- | --- | --- | --- | --- | --- |
| Wood pigeon | *Columba palumbus* | Cpal | farmland / shrubland / oak forest | 2.2 | Gn |
| Turtle dove | *Streptopelia turtur* | Stur | farmland | 1.0 | FA |
| Cuckoo | *Cuculus canorus* | Cucc | shrubland / oak forest | 1.7 | QF |
| Green woodpecker | *Picus viridis* | Pvir | farmland / oak forest | 1.7 | FA |
| Spotted woodpecker | *Dendrocopus major* | Dmaj | oak forest | 1.0 | QF |
| Woodlark | *Lullula arborea* | Larb | shrubland | 1.0 | SH |
| Skylark | *Alauda arvensis* | Aarv | farmland / shrubland | 1.3 | SH |
| Tawny pipit | *Anthus campestris* | Acam | shrubland | 1.0 | SH |
| Tree pipit | *Anthus trivialis* | Atri | farmland / shrubland / oak forest | 2.0 | FA |
| Pied wagtail | *Motacilla alba* | Malb | farmland | 1.0 | FA |
| Dunnock | *Prunella modularis* | Pmod | farmland / shrubland / oak forest | 3.0 | Gn |
| Robin | *Erithacus rubecula* | Erub | farmland / shrubland / oak forest | 2.4 | Gn |
| Black redstart | *Phoenicurus ochruros* | Poch | farmland / shrubland | 1.2 | FA |
| Stonechat | *Saxicola torquatus* | Stor | farmland / shrubland / oak forest | 2.2 | SH |
| Blackbird | *Turdus merula* | Tmer | farmland / shrubland / oak forest | 2.5 | Gn |
| Song thrush | *Turdus philomelos* | Tphi | oak forest | 1.0 | QF |
| Mistle thrush | *Turdus viscivorus* | Tvis | farmland / shrubland / oak forest | 2.9 | Gn |
| Blackcap | *Sylvia atricapilla* | Satr | farmland / shrubland / oak forest | 2.6 | Gn |
| Whitethroat | *Sylvia communis* | Scom | farmland / shrubland / oak forest | 2.7 | Gn |
| Dartford warbler | *Sylvia undata* | Sund | farmland / shrubland / oak forest | 1.5 | SH |
| Melodious warbler | *Hippolais polyglotta* | Hpol | farmland / shrubland / oak forest | 2.7 | Gn |
| Bonelli's warbler | *Phylloscopus bonelli* | Pbon | oak forest | 1.0 | QF |
| Iberian chiffchaff | *Phylloscopus ibericus* | Pibe | farmland / shrubland / oak forest | 2.1 | QF |
| Firecrest | *Regulus ignicapilla* | Rign | farmland / shrubland / oak forest | 1.4 | QF |
| Wren | *Troglodytes troglodytes* | Ttro | farmland / shrubland / oak forest | 2.7 | Gn |
| Great tit | *Parus major* | Pmaj | farmland / shrubland / oak forest | 2.2 | Gn |
| Coal tit | *Parus ater* | Pate | farmland / shrubland / oak forest | 1.4 | QF |
| Blue tit | *Parus caeruleus* | Pcae | farmland / shrubland / oak forest | 2.0 | QF |
| Crested tit | *Parus cristatus* | Pcri | farmland / shrubland / oak forest | 1.9 | QF |
| Long-tailed tit | *Aegithalos caudatus* | Acau | farmland / oak forest | 1.7 | QF |
| Nuthatch | *Sitta europaea* | Seur | oak forest | 1.0 | QF |
| Short-toed treecreeper | *Certhia brachydactyla* | Cbra | farmland / shrubland / oak forest | 1.3 | QF |
| Jay | *Garrulus glandarius* | Ggla | farmland / shrubland / oak forest | 2.1 | QF |
| Spotless starling | *Sturnus unicolor* | Suni | farmland / shrubland / oak forest | 1.3 | FA |
| Oriole | *Oriolus oriolus* | Oori | farmland / oak forest | 1.9 | QF |
| House sparrow | *Passer domesticus* | Pdom | farmland | 1.0 | FA |
| Chaffinch | *Fringilla coelebs* | Fcoe | farmland / shrubland / oak forest | 2.1 | QF |
| Linnet | *Carduelis cannabina* | Ccan | farmland / shrubland / oak forest | 2.2 | SH |
| Greenfinch | *Carduelis chloris* | Cchl | farmland / oak forest | 1.8 | FA |
| Serin | *Serinus serinus* | Sser | farmland / shrubland / oak forest | 1.6 | FA |
| Bullfinch | *Pyrrhula pyrrhula* | Ppyr | farmland / shrubland / oak forest | 1.5 | QF |
| Yellowhammer | *Emberiza citrinella* | Ecit | farmland | 1.0 | FA |
| Rock bunting | *Emberiza cia* | Ecia | farmland / shrubland / oak forest | 2.2 | SH |

For each species is indicated the species code, land-uses where the species was recorded, habitat breadth and species affinity group: FA – farmland species, SH – shrubland species, QF – forest species, Gn – generalist species.
